# Chronic electrical stimulation with a peripheral suprachoroidal retinal implant: a preclinical safety study of neuroprotective stimulation

**DOI:** 10.3389/fcell.2024.1422764

**Published:** 2024-06-20

**Authors:** Carla J. Abbott, Penelope J. Allen, Chris E. Williams, Richard A. Williams, Stephanie B. Epp, Owen Burns, Ross Thomas, Mark Harrison, Patrick C. Thien, Alexia Saunders, Ceara McGowan, Caitlin Sloan, Chi D. Luu, David A. X. Nayagam

**Affiliations:** ^1^ Centre for Eye Research Australia, Royal Victorian Eye and Ear Hospital, East Melbourne, VIC, Australia; ^2^ Department of Surgery (Ophthalmology), University of Melbourne, East Melbourne, VIC, Australia; ^3^ Vitreoretinal Unit, Royal Victorian Eye and Ear Hospital, East Melbourne, VIC, Australia; ^4^ Bionics Institute, East Melbourne, VIC, Australia; ^5^ Medical Bionics Department, University of Melbourne, Fitzroy, VIC, Australia; ^6^ Department of Clinical Pathology, University of Melbourne, Parkville, VIC, Australia; ^7^ Dorevitch Pathology, Heidelberg, VIC, Australia

**Keywords:** retinal implant, neuroprotection, electrical stimulation, electroretinography, retinal histopathology, surgical feasibility

## Abstract

**Purpose:**

Extraocular electrical stimulation is known to provide neuroprotection for retinal cells in retinal and optic nerve diseases. Currently, the treatment approach requires patients to set up extraocular electrodes and stimulate potentially weekly due to the lack of an implantable stimulation device. Hence, a minimally-invasive implant was developed to provide chronic electrical stimulation to the retina, potentially improving patient compliance for long-term use. The aim of the present study was to determine the surgical and stimulation safety of this novel device designed for neuroprotective stimulation.

**Methods:**

Eight normally sighted adult feline subjects were monocularly implanted in the suprachoroidal space in the peripheral retina for 9–39 weeks. Charge balanced, biphasic, current pulses (100 μA, 500 µs pulse width and 50 pulses/s) were delivered continuously to platinum electrodes for 3–34 weeks. Electrode impedances were measured hourly. Retinal structure and function were assessed at 1-, 2-, 4-, 6- and 8-month using electroretinography, optical coherence tomography and fundus photography. Retina and fibrotic thickness were measured from histological sections. Randomized, blinded histopathological assessments of stimulated and non-stimulated retina were performed.

**Results:**

All subjects tolerated the surgical and stimulation procedure with no evidence of discomfort or unexpected adverse outcomes. The device position was stable after a post-surgery settling period. Median electrode impedance remained within a consistent range (5–10 kΩ) over time. There was no change in retinal thickness or function relative to baseline and fellow eyes. Fibrotic capsule thickness was equivalent between stimulated and non-stimulated tissue and helps to hold the device in place. There was no scarring, insertion trauma, necrosis, retinal damage or fibroblastic response in any retinal samples from implanted eyes, whilst 19% had a minimal histiocytic response, 19% had minimal to mild acute inflammation and 28% had minimal to mild chronic inflammation.

**Conclusion:**

Chronic suprathreshold electrical stimulation of the retina using a minimally invasive device evoked a mild tissue response and no adverse clinical findings. Peripheral suprachoroidal electrical stimulation with an implanted device could potentially be an alternative approach to transcorneal electrical stimulation for delivering neuroprotective stimulation.

## 1 Introduction

Degenerative retinal diseases are a global health issue. Despite current management strategies, retinitis pigmentosa (RP), age-related macular degeneration (AMD) and glaucoma remain primary causes of blindness and visual impairment, with over 270 million people affected worldwide (Jonas et al., 2017; Allison et al., 2020; Steinmetz et al., 2021; Cross et al., 2022). RP and AMD lead to the progressive death of the light-sensing photoreceptors in the retina, whereas glaucoma causes a progressive loss of the retinal ganglion neurons (Sorrentino et al., 2016; Weinreb et al., 2016; Fleckenstein et al., 2021). RP is the leading cause of inherited vision loss in working age adults (Liew et al., 2014; Heath Jeffery et al., 2021; Cross et al., 2022), while the contributions of glaucoma and AMD to global blindness and irreversible visual impairment increase markedly with age (Steinmetz et al., 2021). AMD alone is projected to rise from 196 million in 2020 to 288 million people by 2040 (Wong et al., 2014). Together, these conditions result in a substantial global health and economic burden (Wong et al., 2014; Steinmetz et al., 2021; Cross et al., 2022).

Currently, the treatments for glaucoma aim to lower intraocular pressure by use of medications (eyedrops or advanced drug delivery systems), laser, or surgery, however, not all patients can achieve successful control of the disease and continue to progress and lose their vision (Weinreb et al., 2016; Yadav et al., 2019; Sheybani et al., 2020). There are intravitreal injections available to improve visual outcomes for late-stage AMD, however there is no curative treatment available to stop progression from earlier stages to late-stage AMD (Guymer and Campbell, 2023). There is one gene therapy (voretigene neparvovec-rzyl; Luxturna) now available for a genetic subtype of retinitis pigmentosa (those with the biallelic RPE65 mutation form of Leber’s congenital amaurosis), that affects about 2% of those with RP (Lloyd et al., 2019; Maguire et al., 2019). However, there are no curative treatments available for the remaining 98% (∼2 million) of people worldwide with RP. There has been great progress in research targeting gene and cell therapies for glaucoma, AMD and RP, however only the single above-mentioned gene therapy is currently approved, although others are in late-stage trials (Drag et al., 2023; Voisin et al., 2023). Critically, not everyone with these conditions will be eligible for gene therapy due to the large genetic heterogeneity underlying all three conditions. Stem cell treatments, primarily aimed at restoring or replacing dysfunctional cells, are still in clinical trials and are not appropriate for treating earlier stage disease when central vision is still present (Voisin et al., 2023). In summary, many people with glaucoma, AMD and RP continue to lose vision after diagnosis, despite accessing optimal care (Sheybani et al., 2020; Guymer and Campbell, 2023). Furthermore, aside from voretigene neparvovec-rzyl (often given in childhood), there are no curative treatments for early-stage retinal disease on the near horizon.

A paradigm-shifting treatment would be to arrest retinal degeneration before patients lose useful vision. Low level electrical stimulation is believed to protect the retina from degeneration (Morimoto, 2012; Pardue et al., 2014). Preclinical model and clinical studies suggest that low level electrical stimulation is effective for retinal and optic nerve diseases such as glaucoma, AMD and RP (Morimoto et al., 2007; Morimoto et al., 2010; Schatz et al., 2011; Bittner and Seger, 2018; Shen et al., 2021; Parkinson et al., 2023; Stett et al., 2023; Li et al., 2024). A systematic review of the clinical literature suggests electrical stimulation has “promising therapeutic effects on RP and optic neuropathy” although indicates that “more large-scale randomized controlled trial studies should be conducted to elucidate the potential of electrical stimulation, especially on AMD, retinal artery occlusion and glaucoma” (Liu et al., 2021). Furthermore, extraocular electrical stimulation devices using transcutaneous or transcorneal stimulation have received marketing approval by regulatory agencies in some jurisdictions. Although these extraocular systems are becoming available, they necessitate setting up electrodes on the eyelid or cornea for often weekly stimulation sessions (Stett et al., 2023). An implantable system has potential advantages by requiring minimal set up (a magnetically-coupled behind-the-ear receiver stimulator only), allowing the person to continue with daily activities in their home environment whilst receiving stimulation, with no side effects of dry eye or ocular irritation.

Hence, in this study we developed a novel neuroprotective eye implant, placed outside the retina in the suprachoroidal location with a simple, minimally invasive surgery. The design leverages experience from developing a retinal prosthesis, that has successfully been implanted in two clinical trials (Ayton et al., 2014; Petoe et al., 2021; Petoe et al., 2024). This approach is compatible with therapeutic retinal stimulation as it does not interfere with natural vision nor evoke overlapping percepts; thus, it can be used in patients with early-stage disease and residual vision. The primary aim of this study was to perform an *in vivo* safety assessment of the novel neuroprotective eye implant to assess surgical safety and chronic low-level stimulation safety in a large-eye pre-clinical model. The study has been designed to provide evidence for proceeding to first-in-human clinical trials with a minimally-invasive neuroprotective implant in patients with progressive vision loss due to conditions such as RP, AMD and glaucoma. The secondary aim of this study was to assess the functionality of the novel preclinical implant stimulation system design in chronic stimulation studies. This part of the study was designed to provide evidence of successful long term chronic stimulation with the system.

## 2 Materials and methods

Similar methodology to published preclinical suprachoroidal retinal prosthesis safety studies was followed (Nayagam et al., 2014; Abbott et al., 2018). However, in the present study, the aim was to assess the effect of therapeutic level electrical stimulation on the peripheral retinal tissue rather than phosphene-evoking stimulus of the central retina. An added component of the present study was to evaluate the form of the implant; trading-off implant length, surface area, and position beneath the retina, with the degree of surgical invasiveness. The safety of chronic continuous electrical stimulation delivered to the retina via an electrode array located in the suprachoroidal space was assessed by monitoring longitudinal electrode impedance, longitudinal retinal structure and function, and terminal histopathological evaluation. Electrode impedances were regularly measured to assess the stability of the electrode-tissue interface. Standard clinical tools (color fundus photography, spectral domain optical coherence tomography [SDOCT] and electroretinography [ERG]) were used to evaluate the structure and function of the retina *in vivo*. At study completion, the retinal tissue was evaluated histopathologically for any evidence of electrical stimulation induced injury including inflammatory response.

### 2.1 Minimally-invasive suprachoroidal array

The minimally invasive retinal-degeneration arrestor (MIRA) array design required a minimally-invasive surgical approach. This involved a suprachoroidal position where the array does not come into direct contact with the retina, which markedly reduces the chance of retinal damage during insertion. Additionally, it required a peripheral location and small size where the array substrate is distant to the central retina to reduce any chance of surgical or stimulation induced damage to the macula in people that still have residual central vision. For these reasons, the MIRA array was designed within a small substrate with only five electrodes compared to the large substrate and 46 electrodes in the bionic eye array (Petoe et al., 2021).

Two MIRA prototype arrays with different substrate sizes (relatively “short” and “long”) were designed for testing within the preclinical feline model. Prototypes of two different sizes were trialed due to the trade-off between ideal size (smaller is better as it is more minimally-invasive) and surgical handling (larger is easier to handle). Furthermore, there are array positioning considerations in terms of the scleral incision position relative to the ora serata. The implant length needed to be greater than the distance from the scleral incision to the ora serrata, but also needed to have stimulating electrodes located as peripheral as possible to meet design criteria of a minimally-invasive device located away from the area centralis. Due to differences in scleral thickness (and hence incision location relative to the limbus) in human and feline eyes, there was a possibility that a “short” design may not extend past the ora serrata in the feline model. Although both designs will generate electric fields able to stimulate the retinal neurons and glia (like the previous extraocular stimulation studies), since the aim was to assess the safety of the implantation and stimulation on the retina (in translation to human use both prototypes would be underlying the retina), it was considered ideal to have at least one prototype located under the retina in the feline model to assess comprehensively for any local effects of stimulation.

**FIGURE 1:**
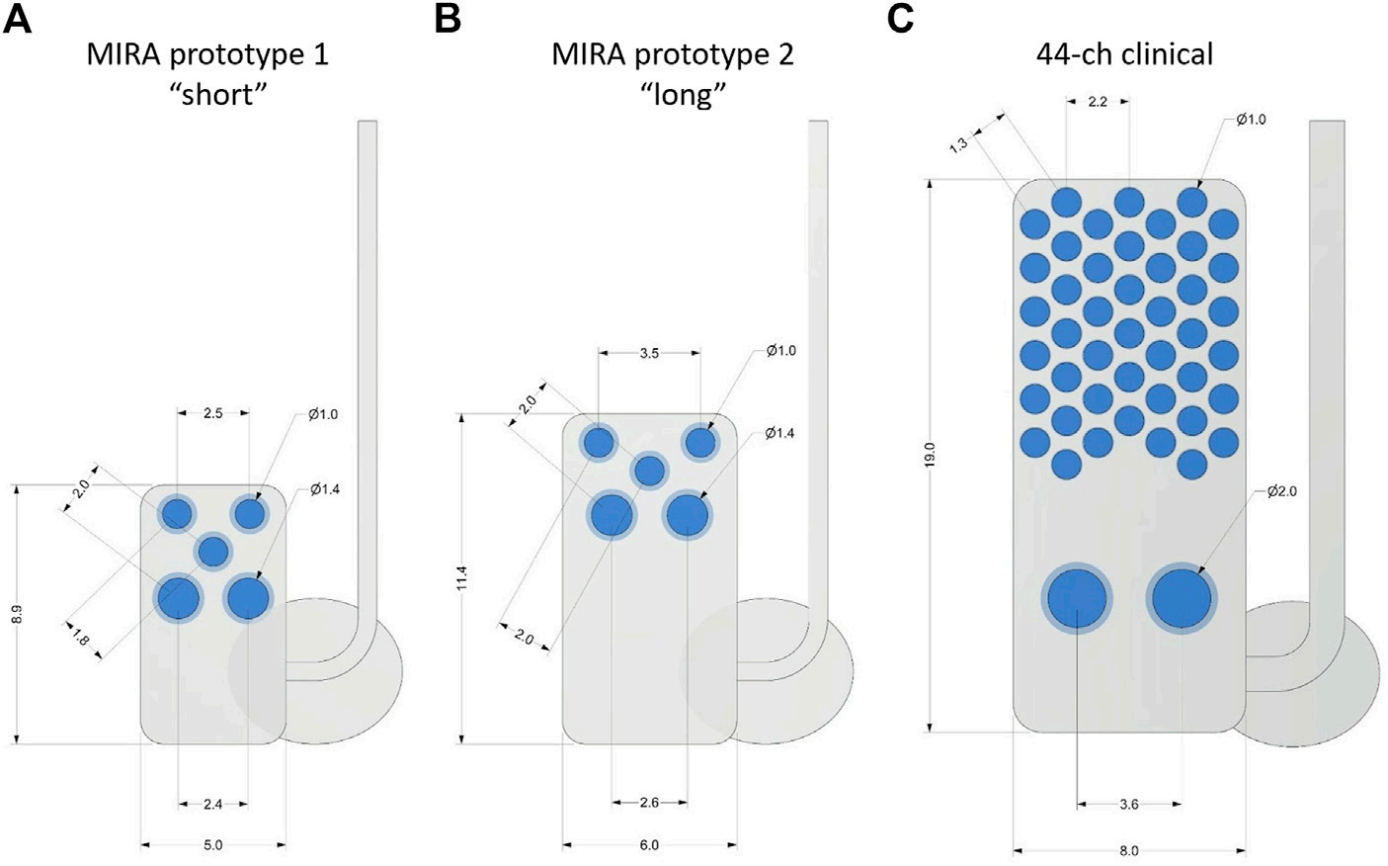
Comparison of suprachoroidal electrode array designs with measurements shown in mm. **(A)** MIRA prototype 1 (“short”), **(B)** MIRA prototype 2 (“long”), and **(C)** the 44-channel electrode array used in the second generation suprachoroidal prosthesis clinical trial. The MIRA prototypes have three x Ø 1.0-mm platinum stimulating electrodes and two x Ø 1.4-mm platinum return electrodes with prototype 1 using a 8.9 × 5.0-mm silicone carrier and prototype 2 using a 11.4 × 6.0-mm silicone carrier. The 44-channel clinical array has 44 x Ø 1.0-mm platinum stimulating electrodes and two x Ø 2.0-mm platinum return electrodes in a 19 × 8.0-mm silicone carrier. The scleral fixation patches were used to anchor the implant to the sclera. Ø, diameter.

The comparison of protype array dimensions to the human bionic eye array is shown in Figure 1. All three designs were manufactured using a medical grade silicone substrate and platinum electrodes, which are known to be well-tolerated by eye tissues and straightforward to remove or replace (Villalobos et al., 2013; Nayagam et al., 2014; Leung et al., 2015; Abbott et al., 2018). The MIRA prototypes have three x Ø 1.0-mm platinum stimulating electrodes and two x Ø 1.4-mm platinum return electrodes. Prototype 1 (“short”) uses a 8.9 × 5.0-mm silicone carrier and prototype 2 (“long”) uses a 11.4 × 6.0-mm silicone carrier. In comparison, the human suprachoroidal retinal prosthesis array has 44 x Ø 1.0-mm platinum stimulating electrodes and two x Ø 2.0-mm platinum return electrodes in a 19 × 8.0-mm silicone carrier. Scleral fixation patches to anchor the implant to the sclera and orbital fixation patches to anchor the cable to the orbit, made of Dacron-reinforced elastomer were used in all three designs. Individually insulated platinum-iridium wires were welded to each electrode in the array and then coiled together into a helical silicone cable with 1.2 mm diameter.

### 2.2 Implant system

**FIGURE 2:**
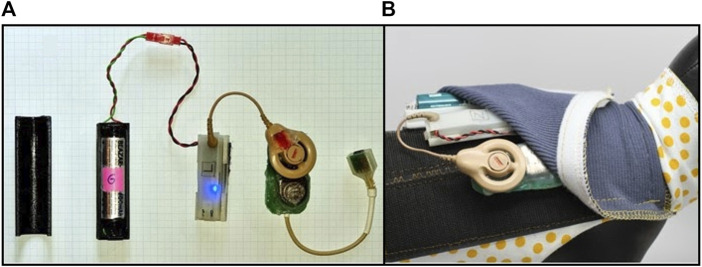
Battery-pack stimulation system in feline model. **(A)** Stimulation hardware from left to right, battery cover, battery and cable, stimulation control system (Nimble) to generate stimulation and measure impedance, Cochlear Implant CI-24RE connected to a circuit board, connector to plug into implant cable, **(B)** Feline backpack designed for strapping around the body (shown on a model) and secured with Velcro and the accompanying stimulation hardware inserted into the pouch of the backpack.

A novel implant system incorporating platinum wires routed all the way through the cable to the terminal (exterior) connector was developed for the feline model. From the orbital patch on the lateral orbital margin, the cable system traverses under the skin to the top of the skull and follows along the back of the skull to exit at a percutaneous extrusion at the neck, where the cable connects to the backpack stimulation system via a semi-customised mini-USB connector (Figure 2).

Within the backpack stimulation system, there are hardware components comprising a battery cover, a battery and cable and a stimulation control system (Nimble) to control stimulation and measure and log impedances (Thien et al., 2018), a Cochlear Implant CI-24RE stimulator to generate voltage controlled biphasic current pulses, connected to the cable wiring via a customized printed circuit board (Figure 2). The backpack system itself is designed for strapping around the body and is secured with Velcro both around the body and around the pouch with the hardware components. The battery requires changing approximately every 2–3 days.

### 2.3 Cohort and timeline

**FIGURE 3:**
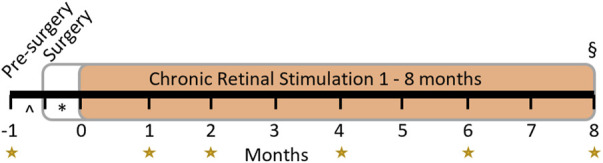
Experimental timeline. Subjects had clinical assessments at regular time points relative to device switch-on (the start of the chronic retinal stimulation period in orange) for up to 8 months as indicated by the yellow stars. The implant surgery occurred >2 weeks (*) prior to switch-on and clinical pre-surgery assessment was performed >2 weeks (^) prior to surgery. Chronic stimulation of the retina began 2 weeks after surgery and continued to endpoint (§, maximum 8 months). If electrode functionality ceased prior to planned endpoint (8-month), clinical evaluation and electrical stimulation ceased, and the endpoint was brought forward. Histopathology was performed at endpoint in all subjects.

Eight normal healthy adult *Felis catus* were used. Each subject’s experiment schedule followed a timeline of procedures and assessments as shown in Figure 3. In summary, each subject underwent clinical assessments of external and internal ocular health at least 2 weeks prior to monocular implantation. Backpack stimulators were activated approximately 2 weeks after surgery. Clinical internal assessments were repeated prior to and regularly after the commencement of chronic stimulation for as long as the system remained functional, out to a maximum of 8-month stimulation. Terminal procedures included transcardial perfusion with both eyes prepared for histopathological evaluation. Full experimental details are outlined below.

### 2.4 Anesthesia and monitoring

The anesthetic and monitoring procedures used in the present study are similar to previously published reports (Villalobos et al., 2013; Nayagam et al., 2014; Abbott et al., 2018). Briefly, for clinical assessments a deep anesthetic state was induced with subcutaneous xylazine (2 mg/kg s. c.; Ilium Xylazil-20, Troy Laboratories, NSW, Australia) and ketamine (20 mg/kg s. c.; Ilium Ketamil, Troy Laboratories, NSW, Australia). For implant surgery, xylazine and ketamine (dosage as above) was used to induce a deep anesthetic state and this was maintained with gaseous Isoflurane (Abbott Australasia, Pty Ltd Australia; 1%–3%) delivered via an endotracheal tube. The subject’s breathing and blood pressure were monitored regularly during all procedures (Cardell veterinary monitor 9,405, Casmed Medical Systems, USA) and body temperature was maintained at 37 °C. Clinical assessments took 1.5–2 h and implant surgeries took 3–5 h. During procedures, pupils were dilated with 1% tropicamide (Chauvin Pharmaceuticals, Surrey, England) and 2.5% phenylephrine hydrochloride (Chauvin Pharmaceuticals, Surrey, England), and subjects were rehydrated with Hartmann’s solution (5 mL/kg/h; s. c.).

### 2.5 Implantation surgery

**FIGURE 4:**
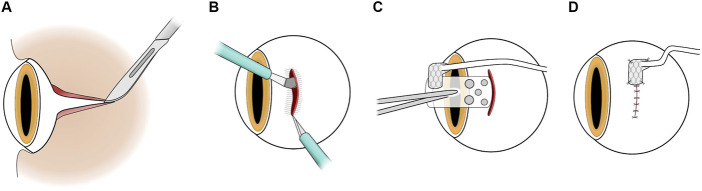
Surgical method for implantation of the suprachoroidal electrode array. **(A)** Canthotomy and conjunctival peritomy. **(B)** Scleral incision and creation of suprachoroidal pocket. **(C)** Electrode array insertion into the suprachoroidal pocket. **(D)** Scleral wound closure and suturing of scleral patch as an anchor-point for the array and the cable.

The surgical procedure for implanting the MIRA array is shown in Figure 4. The cable system has not been previously described, although there are similarities in the array insertion to our previous studies with a retinal prosthesis (Villalobos et al., 2012; Saunders et al., 2014). All surgeries were performed by a vitreoretinal surgeon (PJA) under aseptic conditions. Subjects were deeply anaesthetized, intubated and monitored as above. The planned incision sites were shaved, that is from the lateral canthus to the ear and the superior aspect of the head over the neck to between the scapulae. This area was then prepared with Betadine (Povidone iodine 10% w/v, Alcon Laboratories, Macquarie Park, NSW, Australia) antiseptic solution. Corneal desiccation was prevented with ocular lubricant (HPMC PAA gel; Alcon Laboratories).

Monocular implantation of the device was performed in the left eye for all subjects. A lateral canthotomy was performed and the incision extended towards the ear. A para-midline incision over the scalp was performed, then a transverse skin incision between the scapulae. A subcutaneous dissection was then made between all three incisions. Over the vertex of the skull the muscle and periosteum were pushed aside to reveal the bone. The bony ridge (nuchal crest) at the posterior margin of the skull was drilled to prepare suture anchoring holes on either side of the occipital protuberance. A 360° peritomy was prepared and hemostasis of the episclera/sclera was obtained.

The device was passed from the lateral canthotomy subcutaneously to the vertex of the skull using a trocar. The cable was then externalized via the transverse incision between the scapulae. Sutures were made to periosteum and muscle fascia using 5/0 polyester (Ethicon, Mexico), curved needles (size 18 curved round body, Dolphin Sutures, India) and Dyneema Purity (DSM Biomedical, PA, USA) sutures.

The 6 mm scleral incision began 5 mm from the limbus and was made in parallel with the limbus with a 15° blade (Alcon Laboratories) and a crescent blade (Alcon Laboratories) to dissect the suprachoroidal space and then the electrode array was inserted. The L-shaped extension to the superior margin of the wound was made after the wound was stabilized with 8/0 nylon (Ethicon, Mexico) interrupted sutures, to allow the cable to egress and sit flat on the sclera. 8/0 nylon was then used to suture the Dacron-reinforced silicone patch to the sclera to stabilize the device. After cable stabilization, the implant position was checked by the vitreoretinal surgeon with a binocular indirect ophthalmoscope. The conjunctiva was closed with 8/0 vicryl (Ethicon, Mexico). 5/0 nylon (Ethicon, Mexico) was used to anchor the Dacron-reinforced silicone patch at the orbital margin. The lateral canthotomy was closed in layers with 6/0 vicryl (Ethicon, Mexico) and 5/0 nylon to skin. The vertex wound was closed in layers with 6/0 vicryl and 5/0 nylon and the transverse skin incision with 5/0 nylon.

The wounds were then dressed with Opsite spray dressing (Smith Nephew Medical Ltd, England) and eye drops were administered, g Prednefrin Forte (Phenylephrine hydrochloride; Prednisolone acetate Allergan Australia) and g Chlorsig (chloramphenicol 0.5%; Aspen Pharma PL).

### 2.6 Post-operative care

Subjects were monitored daily throughout recovery period, by staff experienced in animal-husbandry, and were regularly checked by a veterinarian. Analgesic (buprenophrine 0.01 mg/kg, SC; Temgesic; Reckitt Benckiser, Sydney, Australia Temgesic) was administered at the completion of the implantation procedure and again the following day. For the first week post-operatively, the subject was given amoxicillin-clavulanate suspension once daily (10 mg/kg, SC; Clavulox; Pfizer Italia, Rome, Italy). Local and systemic antibiotics (respectively: Chlorsig; Sigma Pharmaceuticals, VIC, Australia; Noroclav; Norbrook, Newry, Northern Ireland), corticosteroids (Predneferin Forte; Sigma Pharmaceuticals, VIC, Australia) and anti-cholinergic drugs (1% atropine sulphate; Chauvin Pharmaceuticals, Surrey, England) were administered as deemed necessary by the surgeons and/or veterinarian. Sutures at the site of the lateral canthotomy, head wound, and cable exit wound were removed under anesthesia at 2-week post-implantation during clinical assessment. The cable exit wound was cleaned and disinfected daily until fibrous encapsulation was achieved (approximately 2–3 weeks), after which it, and the other surgical wounds, were inspected daily and cleaned every few days.

### 2.7 Chronic stimulation

Chronic low-level electrical stimulation with the wearable stimulator system commenced 2 weeks after surgery. Current level was ramped from 50% to 100% (50–100 µA) over 8 days in the pilot subject (17_201). For the other seven subjects, stimulation level was ramped up slowly to 100% on the same day, whilst closely observing subjects to check for any indications of adverse reactions. During electrical stimulation, charge balanced, biphasic, current pulses (100 μA, 500 µs pulse width and 50 pulse/s) were delivered continuously to the three 1.0 mm Ø platinum electrodes. All subjects were stimulated using a monopolar electrode configuration, with the two larger 1.4 mm Ø electrodes designated as the return electrodes. The stimulators received power from a large lithium-ion battery (18,650, 3.7 V). Any electrode found to be of high impedance or out of compliance was excluded from the ongoing stimulation protocol. Extraocular retinal neuroprotection stimulation studies have been successfully performed in humans with retinal or optic nerve disease using current amplitudes of 20–1,000 µA previously, usually for short periods of 30–60 min weekly (Schatz et al., 2011; Bittner and Seger, 2018; Sinim Kahraman and Oner, 2020; Stett et al., 2023). In this study, the subjects received continuous stimulation with the intraocular implant at 100 µA (this is in the range of previous studies) for the entire period of chronic stimulation (up to 8-month).

### 2.8 Electrode impedance measurements

Electrode impedance was measured at the end of the cathodic (first) phase of a biphasic current pulse and defined as the peak voltage divided by the current (John et al., 2011). Although this measurement of ‘impedance’ does not include a phase angle, so could correctly be termed ‘mean of peak cathodic-phase instantaneous resistances’, the term ‘impedance’ has become commonly accepted in many clinical neuroprosthesis studies (Nayagam et al., 2014). Individual electrode impedances were measured wirelessly using custom laboratory software (Nimble) running on a laptop computer (Thien et al., 2018) for all eight subjects. Impedance measurements were calculated and averaged in response to trains of 25 µs phase-width rectangular biphasic current pulses of 75 µA amplitude. Measurements were made intraoperatively to ensure that the implant was functional before closing the surgical wounds. Impedance measurements were then made approximately hourly for the duration of stimulation in each subject. Pre-implantation and post-explanation impedance measurements were made in 0.9% saline. The pre-implantation impedances were measured using different custom software (Timon; 0.5 ms phase-width rectangular biphasic current pulses of 100 µA) for logistical reasons (Senn, 2015; Shepherd et al., 2019). As the Timon and Nimble systems used different parameters, differences in impedance values are expected, noting the purpose of pre-implantation impedance is simply to check electrode functionality prior to surgery.

### 2.9 Clinical assessments

Clinical assessments of retinal function and structure were performed according to the experimental timeline shown in Figure 3. Overall retinal function was assessed using full-field ERG (Espion, Diagnosys LLC, Lowell, MA, USA), to assess for any global changes to retinal health from implantation and stimulation. ERG responses were collected after 20 min of dark adaptation using a range of light flash intensities from 0.01 to 10 cd.s.m^-2^ and recorded simultaneously from both eyes using corneal Jet electrodes (#7506, Fabrinel, La Chaux-de-Fonds, Switzerland) for all eight subjects. In addition, ERG responses to the light flash intensity of 10 cd.s.m^-2^ were also recorded simultaneously using corneal Jet electrodes in both eyes and the suprachoroidal platinum MIRA electrodes in the implanted eye twice, without any adjustment to the corneal electrodes. This enabled comparison of the intra-session response amplitude and reproducibility of the ERG signals between the corneal electrode and the MIRA electrode for six subjects (18_303 to 19_308).

Retinal structure was assessed with color fundus photography (TRC-50Dx, Topcon Medical Systems, NJ, USA) and SDOCT (Spectralis, Heidelberg Engineering GmbH, Heidelberg, Germany) for all eight subjects. Longitudinal color fundus photographs were used to assess retinal health and to determine the position and stability of the implanted array. SDOCT high-resolution line scans were taken across the tip of the implant as well as at the area centralis and optic disc to assess for any localized changes to retinal ultrastructure. Each scan was averaged from 100 frames (ART = 100). Follow-up scan mode was enabled so that electrode position relative to the retina could be tracked over time. In addition to assessment of longitudinal retinal health, the SDOCT data enabled assessment of array conformability to the suprachoroidal space and assessment of whether the array was applying any localized pressure to the choroid.

### 2.10 Termination and tissue preparation

Procedures were conducted as per established protocols (Nayagam et al., 2014; Abbott et al., 2018). Subjects were overdosed with intravenous barbiturate (Pentobarbitone, 150 mg/kg, Ilium, Troy Laboratories Pty Ltd, Australia) and transcardially perfused with intravenous heparin DBL Heparin Sodium injection BP) with warm (37°C) saline and cold (4°C) neutral buffered formalin. Eyes were enucleated along with the implant system, including cable harness and USB connector, which were carefully dissected and post-fixed together with the eyes in Davidson’s fixative, then dehydrated in ethanol. Scleral tissue adjacent to each electrode was identified and dyed to enable the electrode locations to be identified after histological sectioning (Nayagam et al., 2013). Full thickness posterior eye tissue strips were collected to include all electrode-adjacent locations for histological preparation and analyses. Additionally, skin and muscle from the region of the neck adjacent to the cable exit wound was collected as tissue strips for histological analysis.

### 2.11 Histological preparation and retinal and fibrosis thickness measurements

Paraffin embedded serial section (5 µm thick) of tissue adjacent to the neck exit wound of the cable system and of the retina were collected and stained with hematoxylin and eosin. Sections were imaged using an Axio Imager two upright microscope (Carl Zeiss) and Axio Vision software (v 4.8.2, Carl Zeiss). Retinal sections that were cut through dyed regions indicated where adjacent electrodes were located beneath the retinal tissue.

Retinal thickness and fibrous thickness measurements were made from high power digitized photomicrographs using the Axio Vision software using previously described protocol (Nayagam et al., 2014). Briefly, retinal thickness was measured from the retinal pigment epithelium and tapetum junction, to the inner boundary of the retinal nerve fiber layer (inner limiting membrane). Measurements were performed i) in the middle of overlying electrodes, ii) at a location approximately 500 µm adjacent to electrodes, and iii) in an equivalent position in the fellow eye. Fibrous thickness was measured as the distance across the lining of continuous strongly eosin-stained acellular collagen between i) the suprachoroidal space and the choroid, and ii) between the suprachoroidal space and the sclera (i.e., both sides of the electrode array). Measures were taken at the center of each dyed scleral region overlying electrodes as well as at a location approximately 500 µm adjacent to electrodes. Retinal thickness measurements could only be made when electrodes were inserted past the ora serrata so that retina overlying the array was present. As there were five electrodes per implanted eye, there were up to five locations measured per subject per parameter.

### 2.12 Pathology assessments and scoring

Representative samples of hematoxylin and eosin-stained vertical retinal sections were examined microscopically by one pathologist (RAW) and one senior researcher experienced with retinal pathology (DAXN) and graded in a double-blinded procedure for up to five locations overlying electrodes (implanted eye) or at matched topographical positions (fellow eye) per subject. The tissue was graded on a five-point scale (0 = no pathology, 1 = minimal pathology, 2 = mild pathology, 3 = moderate pathology, 4 = severe pathology). Scores were assigned for potential stimulus-induced tissue damage for histiocytic response, fibroblastic response, chronic inflammatory response, acute inflammatory response, scar tissue, insertion trauma, necrosis or retinal damage. Graders were both experienced with the normal trajectory of chronic passive implantation of electrode arrays and used that knowledge as the baseline. Discrepancies in scoring were discussed and consensus reached. Ten per cent of samples were re-tested to ensure intra-grader consistency. Results were unblinded after all scores were gathered.

### 2.13 Statistics

Distribution of electrical impedance over time was analyzed in R (R Core Team 2021) using box plots to show the median and inter-quartile range, with whiskers following the Tukey boxplot definition representing the lowest datum still within the 1.5 interquartile range of the lower quartile and the highest datum still within the 1.5 interquartile range of the upper quartile. Impedances over 20 kOhm were considered open-circuit and removed from analysis.

Electroretinogram data was analyzed in Prism (v5, GraphPad) with 2-way ANOVAs. If the ANOVA reached significance (0.05), then Bonferroni post-hoc tests were also performed to determine in which group pair significance occurred. Comparison of the agreement in a-wave amplitudes between measuring between a MIRA electrode array and a corneal Jet electrode, was performed with a Pearson’s correlation.

Retinal thickness measures were analyzed in Prism using a one-way ANOVA with Bonferroni’s post-hoc test as the data complied with the normal distribution. Fibrous thickness measures were analyzed in Prism using a non-parametric Kruskal-Wallis test with Dunn’s post-hoc test as the data did not comply with the normal distribution. Histopathology scoring was measured as a cumulative assessment and is shown as a stacked count.

## 3 Results

### 3.1 Cohort, stimulation and electrode survival

The individual implant duration, stimulation duration, timepoints of clinical follow-up, and electrode survival (post explant compliance) at endpoint is shown for each subject at Table 1. The eight subjects were implanted for an average of 6.9 ± 2.3 months (range 2.3–9.8 months) and received electrical stimulation for a period of 4.7 ± 2.9 months (range 0.8–8.7 months). The first subject was a pilot (18_301) and received only 0.8 months of stimulation, whereas the remaining seven subjects received between 1.6 and 8.7 months of stimulation. Notably, due to the new design of the implant cable system, the subjects were able to complete longer duration implantation and chronic stimulation than during previous preclinical chronic studies of the bionic eye device (Villalobos et al., 2013; Nayagam et al., 2014; Abbott et al., 2018). Three subjects received the prototype 1 “short” array, whilst five subjects received the prototype 2 “long” array prototype. Stimulation levels were gradually ramped up for each subject to ensure comfort. A non-pain-related physical response was noted for each subject, indicating that a visual phosphene had been noticed and hence the stimulation was likely above visual threshold. Normal feline behavior, including climbing and jumping, continued during the entire course of chronic stimulation, indicating the initial physical response to stimulation was not related to pain or discomfort. During the course of the study, as electrode channels failed, they were disconnected from the stimulator. At endpoint, 38% of stimulating electrodes in total (9 of 24) remained in compliance. Electrode channel failures were attributed to the percutaneous cable system rather than due to failures within the array itself, as established during post explant microscopy. Reference to stimulating electrodes includes all electrodes that received any stimulation during the study. The endpoint procedures (planned for after 8 months of stimulation) were brought forward if all three stimulating electrodes became open circuit. Open circuit electrodes were discovered during routine impedance testing (Table 1).

**TABLE 1:** The implantation, stimulation and clinical follow up regime for each subject.

Subject	Array	Implant duration (months)	Stimulation duration (months)	Clinical follow up post-stimulation (months)	Electrodes (E) in compliance post explant	Electrodes (E) out of compliance post explant
18_301 (pilot)	Short	2.3	0.8	1		E1, E2, E3
18_302	Short	6.9	5.9	1, 2, 6	E1, E2, E3	
18_303	Long	9.8	8.7	1, 4, 6, 8	E1, E2	E3
18_304	Long	6.3	2.8	2, 4, 6		E1, E2, E3
18_305	Long	7.9	5.1	1, 2, 4, 6	E3	E1, E2
19_306	Short	6.5	1.6	1, 2, 4, 6		E1, E2, E3
19_307	Long	9.0	8.4	1, 2, 4, 6, 8	E1, E2, E3	
19_308	Long	6.3	4.5	1, 2, 4, 6		E1, E2, E3
*Average*	*NA*	*6.9 ± 2.3(SD)*	*4.7 ± 2.9(SD)*	*NA*	*38% of total*	*62% of total*

### 3.2 Array location relative to the ora serrata

**FIGURE 5:**
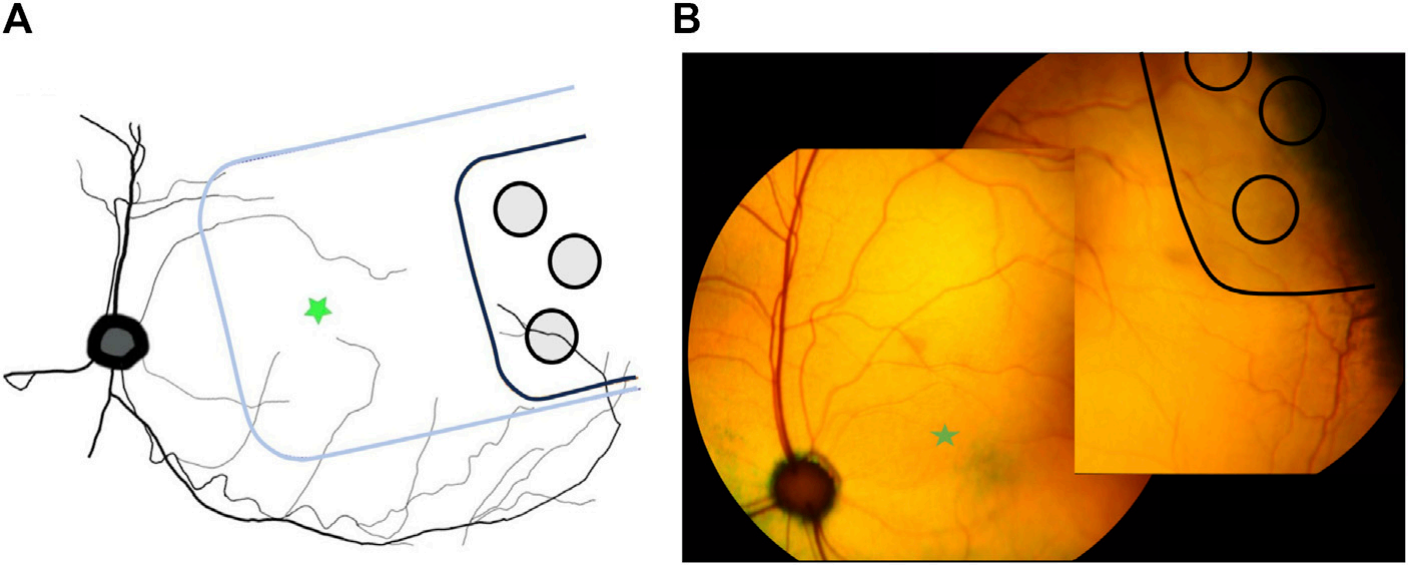
Location of MIRA array in peripheral retina. **(A)** Schematic diagram showing the peripheral location of the MIRA array (black) compared to the usual position of the previously-designed suprachoroidal bionic eye array substrate (blue) under the area centralis. MIRA stimulating electrodes are shown in grey. Green star indicates area centralis. **(B)** Color fundus photos stitched together to show the representative position of the MIRA array (black with light grey shadow) in one subject (19_307), used to generate panel **(A)**. The array is peripheral to the area centralis (green star).

Histology sectioning of the three eyes with prototype 1 “short” arrays, showed that the short arrays did not extend far enough across the ora serrata to have stimulating electrodes overlying retinal tissue. Hence, later subjects were allocated prototype 2 “long” arrays to ensure that retinal health after chronic stimulation could be assessed. Figure 5 shows the location of a prototype 2 “long” MIRA array in peripheral retina relative to the position of a centrally-located suprachoroidal retinal prosthesis array mapped relative to the optic nerve position from a previous feline study (Abbott et al., 2018). The retinal prosthesis array is required to be positioned directly under the area centralis/macula for the purposes of vision restoration, while it is imperative that an array designed for neuroprotection is peripheral to remove any risk of macula damage from surgery.

### 3.3 Surgical recovery and overall health

**FIGURE 6:**
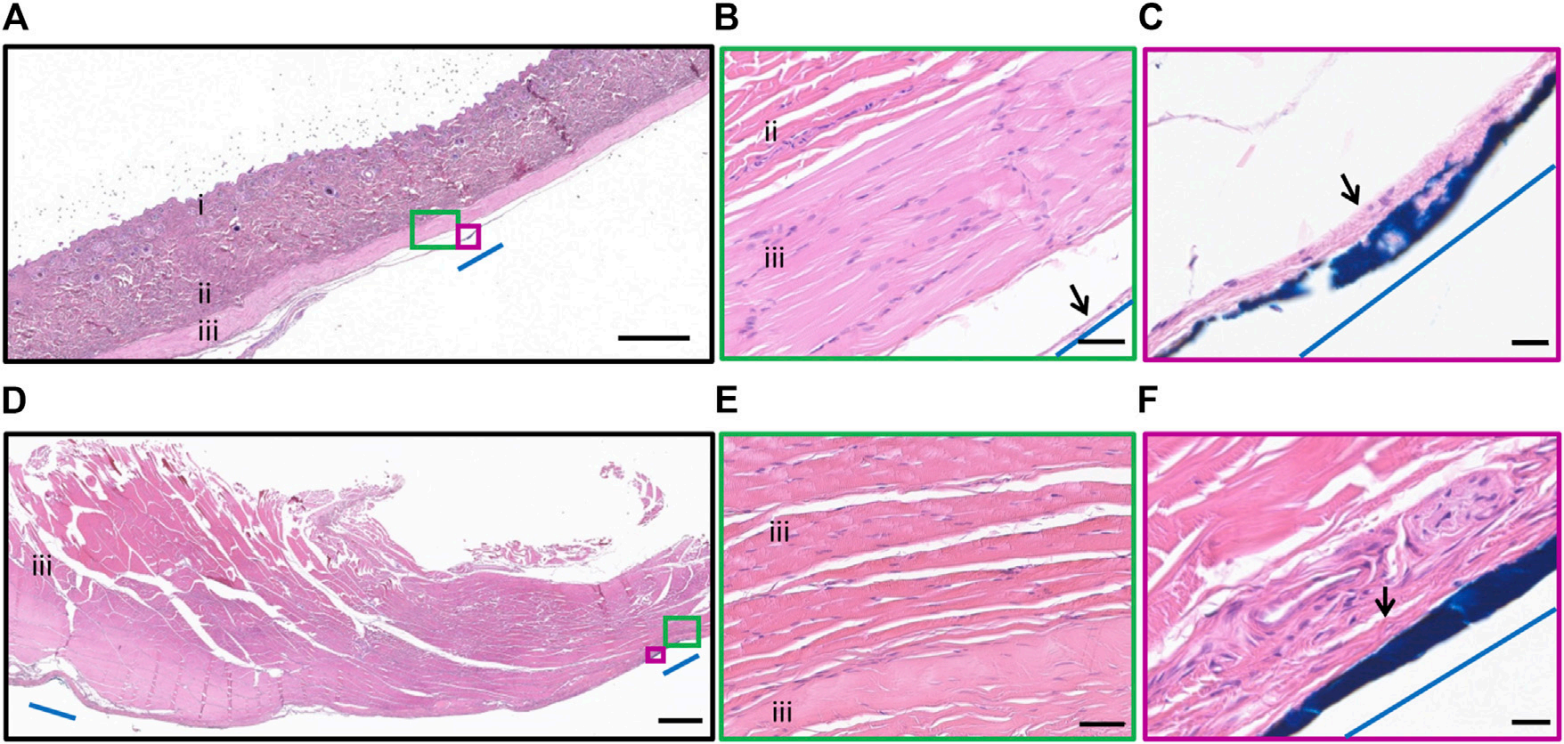
Histopathology surrounding the cable at the neck. Photomicrograph of hematoxylin and eosin stained 5 µm thick paraffin embedded tissue section illustrating the tissue adjacent to the cable at the neck. **(A)** Epidermis and dermis (i), collagen of the subcutaneous layer (ii) and muscle layer (iii) overlying the cable. The cable location was dyed blue dye and indicated by blue line. Scale bar = 1,000 µm. **(B)** Minimal inflammatory fibrotic encapsulation (black arrow), collagen (ii) and muscle layer (iii) adjacent to the cable (blue line). The muscle and collagen appear normal. Scale bar = 50 µm. **(C)** Minimal inflammatory fibrotic encapsulation (black arrow) surrounded the cable (blue dye, blue line). Scale bar = 20 µm. **(D)** Muscle (iii) overlying the head region. The edges of the routing system was dyed with blue dye and indicated by blue line. Scale bar = 1,000 µm. **(E)** Muscle (iii) tissue adjacent to the cable appears normal. Scale bar = 50 µm. **(F)** Minimal inflammatory fibrotic encapsulation (black arrow), adjacent to the routing system at the head (blue dye, blue line). Scale bar = 20 µm.

All subjects recovered well post-operatively and there was no loss of weight in any subject over time. There were no eye infections recorded, however four of eight subjects (18_301, 18_304, 19_306, 19_308) rubbed their orbital region (overlying the cable) and developed short-term mild cutaneous abrasions superior to the eye that were monitored but did not require treatment. Three of eight subjects developed head wounds and localized infections (18_303, 18_304, 18_306) that resolved with short term antibiotics. No other device-related health events were recorded, and all subjects completed the study according to protocol. A photomicrograph of the hematoxylin and eosin stained 5 µm thick paraffin embedded tissue section at the site of the exit wound from the neck (19_307) is shown in Figure 6. There was minimal inflammatory fibrotic capsule formation around the cable as expected. Furthermore, the muscle and other tissue adjacent to the cable appears normal with no atrophy, despite the exit wound.

### 3.4 Electrode impedances

**FIGURE 7:**
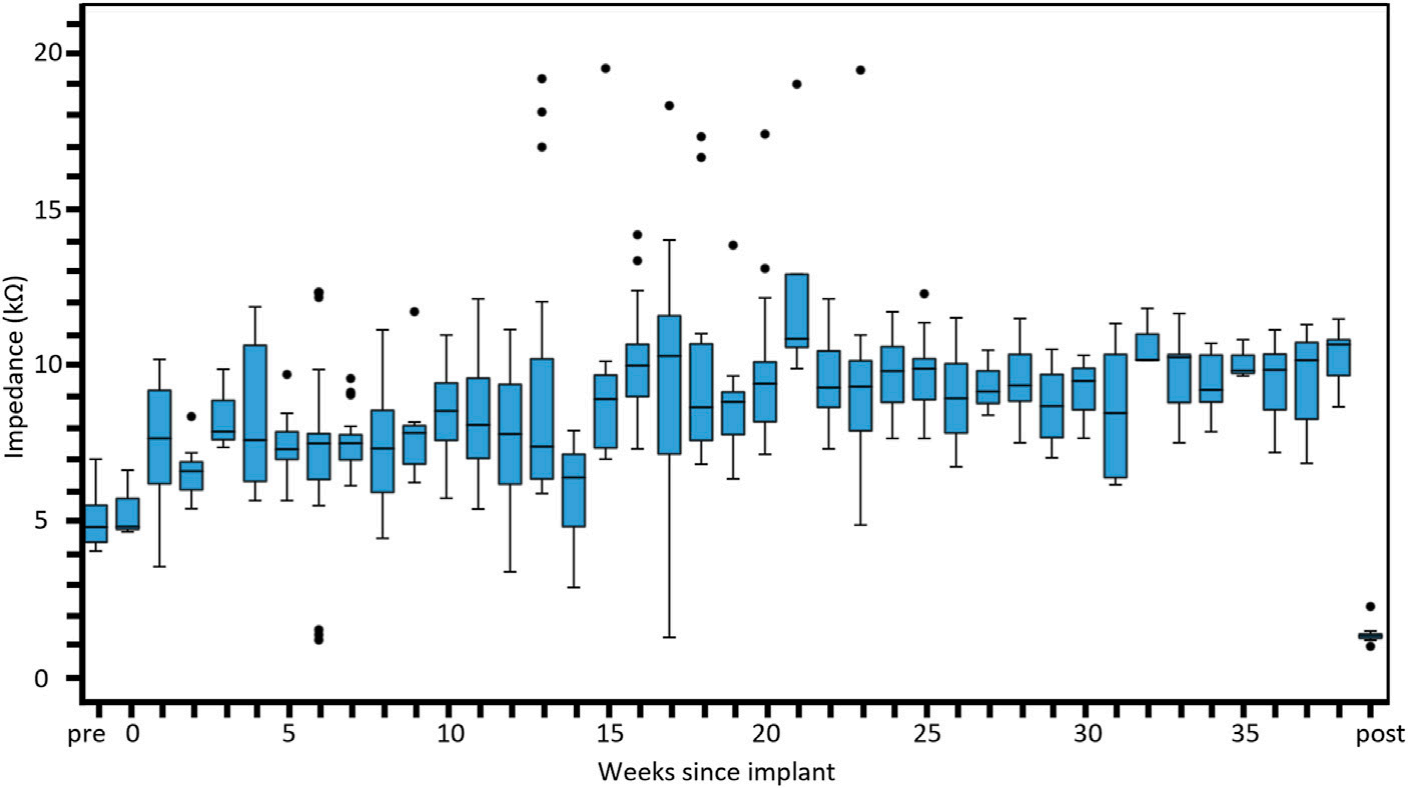
Distribution of electrical impedance over time. Electrode impedance (kΩ) shown across all subjects recorded over the duration of the implantation period up to 8 months. Box plots show the median, first and third quartiles (box edges), while whiskers follow the Tukey boxplot definition representing the lowest datum still within the 1.5 interquartile range of the lower quartile and the highest datum still within the 1.5 interquartile range of the upper quartile. Any points falling outside this range are shown as outliers (black dots). Normal saline was used to perform pre-implantation and post-explantation impedance measurements and expected to be lower than measures in tissue. The number of individual electrode measurements within each box plot ranges from three to 26. The pre-implantation measures were performed with a different measurement system (Timon) to the other measures (Nimble).

Electrode impedance data from the eight active chronic subjects stimulated for up to 8.7 months (36 weeks) are shown in Figure 7. The measured impedances remained reasonably stable ranging between 6 and 10 kΩ in tissue with a small trend for increasing over time. Pre-implantation and post-explantation impedances were less (pre-implant ∼4.5 kΩ, post-explant ∼2.0 kΩ) than impedances measured in tissue (*in vivo*) because they are measured in saline (not tissue), and due to technical necessity since a different system was used for the pre-implantation impedances. Regardless, it is evident that the electrodes were functional prior to surgery, and that even though 62% of electrodes were open circuit at end-point, the remaining functional electrodes had stable impedances. Furthermore, the impedance results in saline pre-implant and post-explant are equivalent to those seen in our previous chronic study (Nayagam et al., 2014).

### 3.5 Array stability and retinal health

**FIGURE 8:**
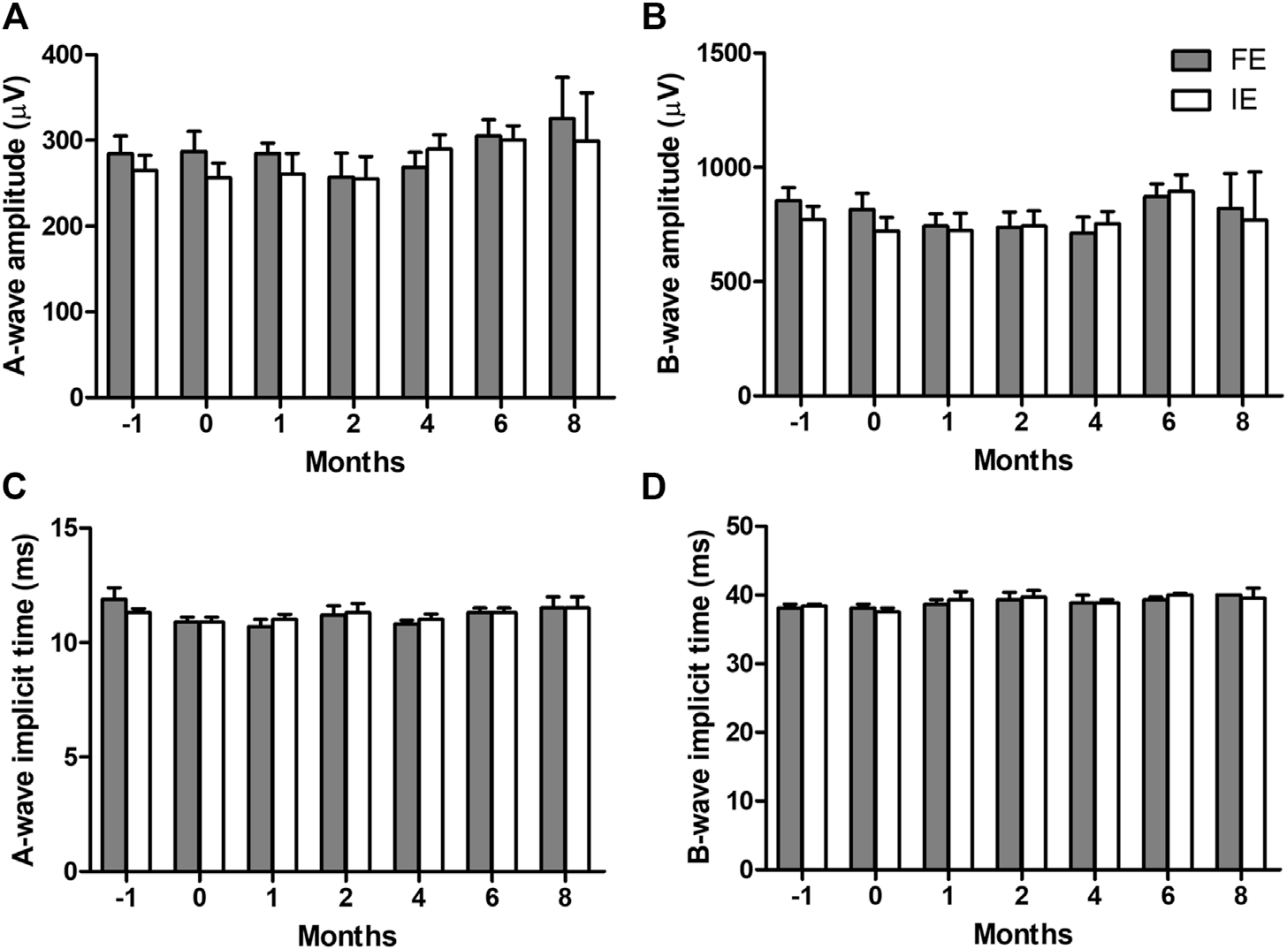
Combined rod-cone maximal full-field electroretinography (ERG) responses longitudinally over the 8 months of chronic electrical stimulation. **(A)** A-wave amplitudes, **(B)** B-wave amplitudes, **(C)** A-wave implicit time, and **(D)** B-wave implicit time in the implanted eye (IE) and fellow eye (FE). 2-way ANOVAs show no difference in any parameter over time (A-wave amplitude *p* = 0.2613, B-wave amplitude *p* = 0.394, A-wave implicit time *p* = 0.161, B-wave implicit time *p* = 0.188) or between implanted and fellow eyes (A-wave amplitude *p* = 0.347, B-wave amplitude *p* = 0.539, A-wave implicit time *p* = 1.00, B-wave implicit time *p* = 0.754). This indicates retinal function remains normal after chronic implantation and retinal stimulation. N = 8 subjects at time −1 month (pre-surgery), N = 8 subjects at time 0 (post-surgery), N = 7 subjects at 1-month (post-stimulation), N = 6 subjects at 2-month, N = 6 subjects at 4-month, N = 6 subjects at 6-month, N = 2 subjects at 8-month. Subjects available at each timepoint are shown in Table 1. Error bars = standard error of the mean.

The combined rod-cone maximal full-field ERG response showed there was no change in global retinal function after array implantation or longitudinally over the 8 months of chronic electrical stimulation (Figure 8). The results were consistent across the A-wave amplitude (*p* = 0.261), A-wave implicit time (*p* = 0.161), B-wave amplitude (*p* = 0.394) and B-wave implicit time (*p* = 0.188). There were also no differences between implanted and fellow eyes (A-wave amplitude *p* = 0.347, A-wave implicit time *p* = 1.00, B-wave amplitude *p* = 0.539, B-wave implicit time *p* = 0.754). Data from all eight subjects was utilized, however, as shown in Table 1, only two subjects made it to the 8-month timepoint.

**FIGURE 9:**
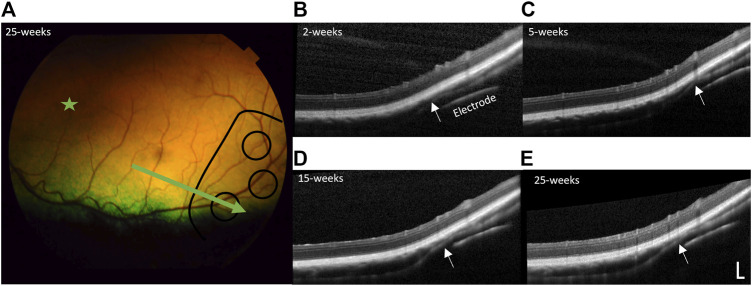
Retinal health over time. **(A)** Representative color fundus photo of far temporal retina 25 weeks post-surgery (23 weeks post-stimulation), with the position of the electrode array outlined in black lines and position of B-scan indicated by the green arrow (subject 19_308). The area centralis is indicated by the green star. Representative longitudinal follow-up spectral domain optical coherence tomography (SDOCT) B-scans through a stimulating electrode and overlying retina at: **(B)** 2-week post-surgery, prior to stimulation, **(C)** 5-week post-surgery (3-week post-stimulation), **(D)** 15-week post-surgery (13-week post-stimulation), and **(E)** 25-week post-surgery (23-week post-stimulation). There is no damage to the retina from surgery or stimulation over time. There is some indentation of the choroid (white arrows) near the tip of the array, remaining constant over time and similar to that reported in retinal prosthesis studies (Abbott et al., 2018) and is not expected to affect blood flow. The array shows mild lateral movement that settles within 15-week of surgery, presumably as the fibrotic capsule forms around the array. SDOCT scale bar = 200 µm.

In all subjects there was no damage to the retina from surgery or stimulation over time, including no folds or tears. In the subjects with the prototype 1 “short” arrays, the 50° field-of-view with the color fundus camera was not wide enough to visualize the implant. Figure 9 shows a color fundus photo and longitudinal SDOCT scans in a representative subject with a prototype 2 “long” array (19_308). Mild indentation of the choroid near the array tip is visible and remains constant over time. This localized indentation is not expected to cause any changes to choroidal blood flow. Mild lateral movement of the array settled within 15-week of surgery, presumably as the expected fibrotic capsule forms around the array to stabilize it.

### 3.6 Electroretinography recordings with MIRA array

**FIGURE 10:**
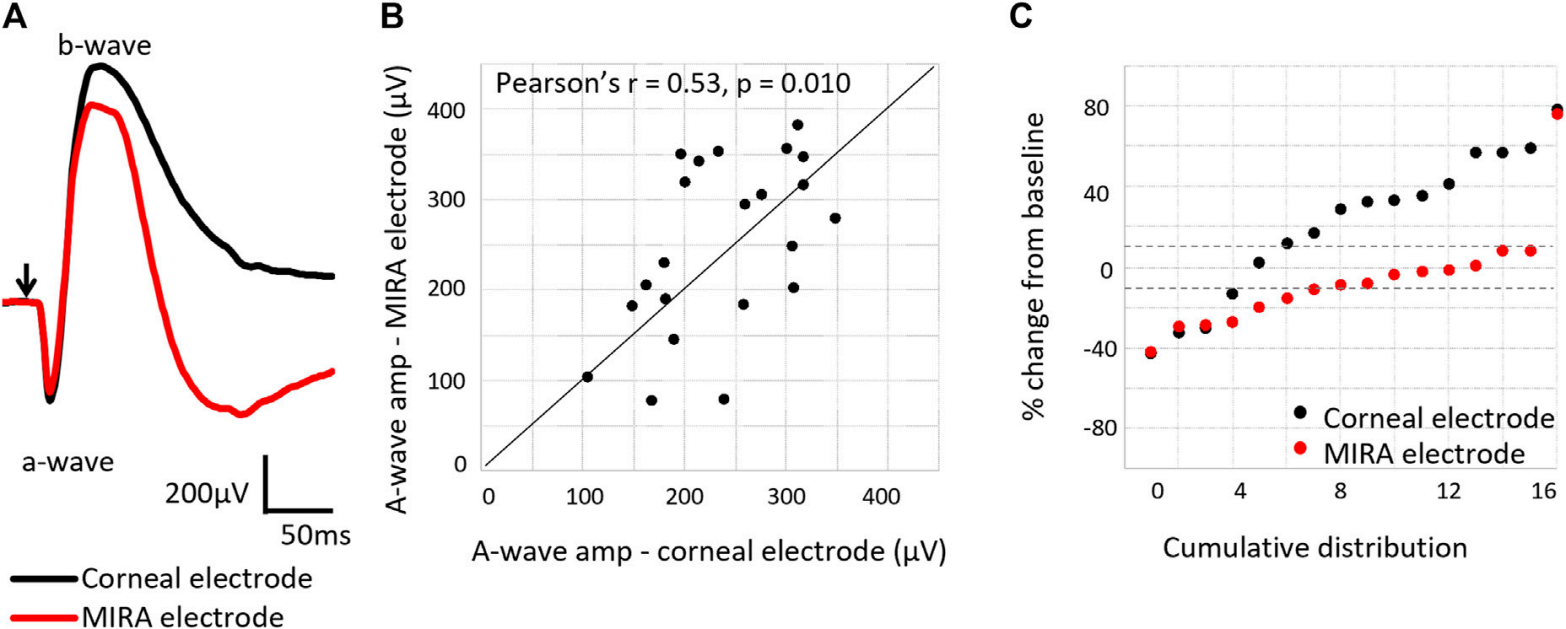
Comparison of electroretinogram response using the corneal Jet electrode compared to the MIRA electrode array. **(A)** Representative electroretinography waveforms (subject 18_302), showing the MIRA electrodes produce a similar electroretinography response to the corneal electrode, except with reduced amplitudes. Arrow indicates light stimulus onset. **(B)** Comparison of the a-wave amplitudes as measured with a MIRA electrode array and a corneal Jet electrode, showing agreement in the measurement (Pearson’s r = 0.53, *p* = 0.010) between the electrodes. N = 24 measurements from six subjects (18_302 to 19_308). **(C)** Percentage change in the a-wave amplitude from baseline measured with the corneal Jet electrodes and MIRA electrode array, showing the MIRA electrode array measurement of retinal function is less variable over time. Dashed lines show ± 10% change from baseline. The majority of the points measured with the MIRA electrode array are within 10% of variation, but this is not the case for the points measured with the corneal Jet electrode.

Simultaneous ERG recordings using both corneal Jet electrodes and the suprachoroidal MIRA electrodes in the implanted eye showed the measurement of retinal function with MIRA electrode array was less variable over time than the corneal Jet electrodes (Figure 10). The MIRA electrodes produced a similar response to the corneal electrodes, except with an expected smaller amplitude due to the placement of the MIRA recording electrode. Comparing the a-wave amplitudes showed a moderate correlation (Pearson’s r = 0.53, *p* = 0.010) between the corneal and MIRA electrodes. Whilst the intra-session reproducibility data showed that percentage change in a-wave amplitude from baseline shows the MIRA array is less variable or more reproducible than the corneal electrode over time.

### 3.7 Retinal and fibrous thickness measurements

**FIGURE 11:**
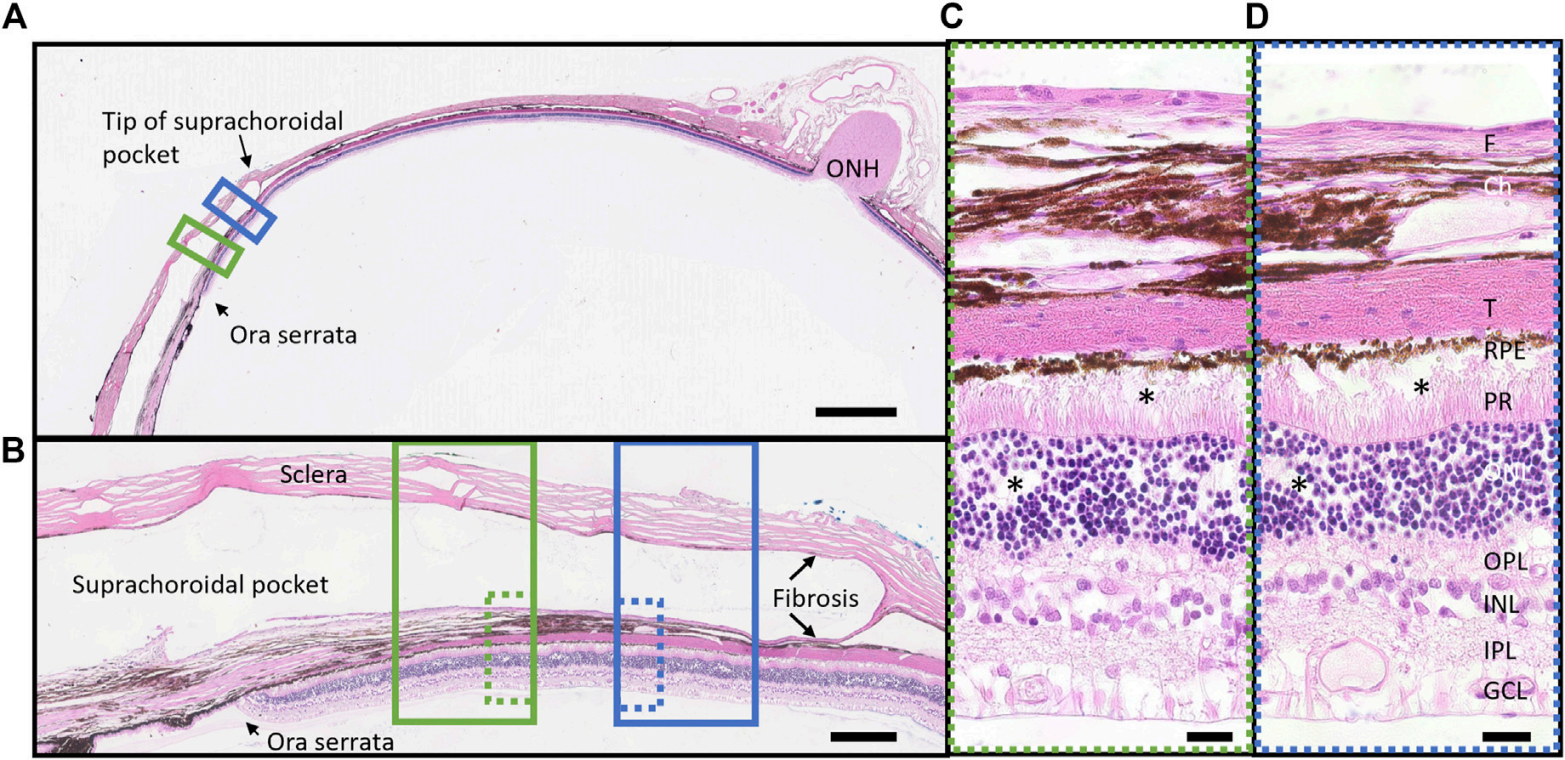
Representative retinal histological staining from a region overlying the electrode (green box) and a region adjacent to electrode (blue box) in a stimulated eye (18_304_OS; long array). **(A)** Overview section demonstrating position of the space occupied by the electrode array (suprachoroidal pocket) in the peripheral retina near the ora serrata. Scale bar = 2000 m. **(B)** High resolution (x20) photomicrograph of the suprachoroidal pocket region, showing retina and sclera overlying an electrode (solid green box, dye over electrode position visible) and at 500 m adjacent to the electrode edge (solid blue box). There is minimal fibrosis (fibrotic capsule) on both sides of the electrode array (scleral and choroidal sides) both overlying and adjacent to an electrode. Scale bar = 250 m. **(C)** Ultra-high resolution (x100 oil) photomicrograph of the retina overlying an electrode (dotted green box), showing no abnormalities after 5.7 months of stimulation. There is minimal fibrosis. There is minor artefact from the microtome at the photoreceptor to RPE boundary (*). Scale bar = 20 m. **(D)** Ultra-high resolution (x100 oil) photomicrograph of the retina 500 m adjacent to the electrode (dotted blue box) showing no abnormalities. There is minimal fibrosis. There is minor artefact from the microtome at the photoreceptor to RPE boundary and in the ONL (*). Scale bar = 20 m. Abbreviations: GCL, ganglion cell layer; IPL, inner plexiform layer; INL, inner nuclear layer; OPL, outer plexiform layer; ONL, outer nuclear layer; PR, photoreceptors; RPE, retinal pigment epithelium; T, tapetum; Ch, choroid; F, fibrosis; ONH, optic nerve head.

A representative histological hematoxylin and eosin-stained vertical section from a region overlying the electrode and a region 500 µm adjacent to the electrode, both within the suprachoroidal “pocket” is shown in Figure 11. There were no retinal abnormalities, and minimal fibrosis. The fibrosis that does form is expected and plays an important role in holding the array in place.

For retinal thickness (inner limiting membrane to retinal pigment epithelium), only subjects with prototype 2 “long” arrays could be quantified, as the prototype 1 “short” arrays did not extend past the ora serrata and could not be captured on OCT. Furthermore, retinal thickness measurements were excluded from two subjects implanted with the long array due to artifactual retinal detachment seen on sectioning. Hence, nine measures of retinal thickness overlying nine electrodes from three subjects with long arrays (18_303, 18_304, 19_307) were available. For fibrosis quantification, both prototype 2 “long” and prototype 1 “short” arrays could be used, as well as eyes with an artifactual retinal detachment due to sectioning procedures, so 33 measures (out of a maximum of 35 possible electrodes to measure) across seven subjects (excludes the pilot subject 18_301) were included.

**FIGURE 12:**
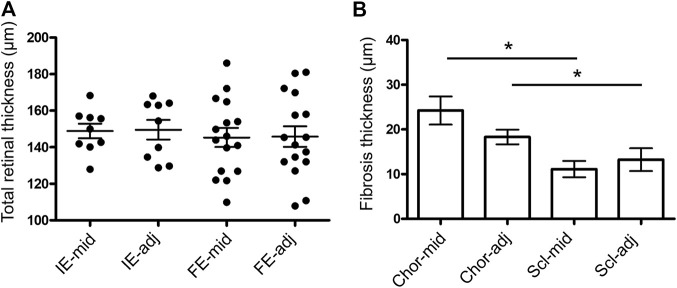
Measures of retinal thickness and fibrosis overlying and adjacent to the electrodes. **(A)** Total retinal thickness (ILM to RPE) measured overlying the middle of each electrode (mid) and 500 µm adjacent to the edge of each electrode (adj) in the implanted eyes (IE; N = 9 measurements). Measurements at matching retinal locations were also taken in the fellow eyes (FE; N = 16 measurements). In implanted eyes, measurements were only able to be taken in eyes with long arrays, as the electrodes in the short arrays did not lie under the retina. One-way ANOVA test (*p* = 0.9362) with Bonferroni’s post-hoc (all comparisons *p* > 0.05). **(B)** Fibrosis thickness on the choroidal (Chor; N = 33 measurements) and scleral (Scl; N = 30 measurements) sides of each electrode overlying the middle (mid) and 500 µm adjacent to the edge of each electrode (adj) in the implanted eyes. Non-parametric Kruskal-Wallis test (*p* < 0.0001) with Dunn’s post-hoc (*p < 0.05 comparisons indicated in graph). In both graphs, a maximum of five measures were taken per eye per subject (N = 7 subjects, excludes pilot 18_301). Error bars both graphs = standard error of the mean.

The quantification of retinal thickness and fibrosis overlying and 500 µm adjacent to the electrodes in implanted and fellow eyes are shown in Figure 12. There was no difference in retinal thickness overlying the middle of electrodes of the implanted eyes to the fellow eyes (matched positions) or in retinal thickness overlying the middle of electrodes to a location 500 µm adjacent within the implanted eyes (*p* = 0.904). Hence, there was no local or global effect of stimulation on retinal thickness. There was a difference in fibrosis thickness between locations (*p* < 0.0001), with the choroidal side of the fibrotic capsule thicker than the scleral side of the fibrotic capsule for measurements both overlying the middle of the electrode and 500 µm adjacent to the edge of electrodes (*p* < 0.05 post-hoc). However, there was no difference in fibrosis thickness between positions overlying the electrode and positions adjacent to the electrode on both sides of the implant (*p* > 0.05 post-hoc). The fibrosis was thickest at the middle of electrodes on the choroidal side, but still only measured at a maximum of 24 ± 18 µm thick at that location.

### 3.8 Retinal histopathology scoring

**FIGURE 13:**
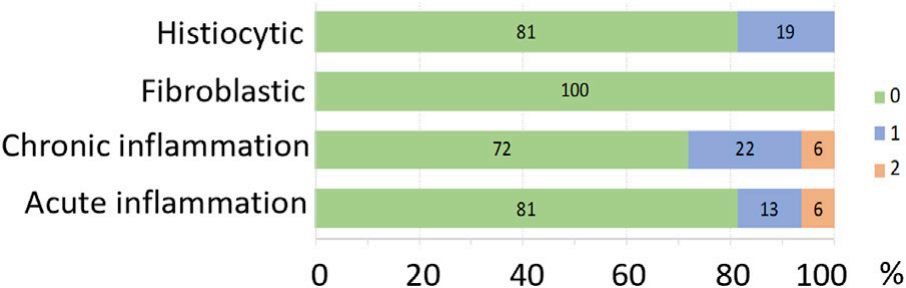
Histopathology grading represented as percentage of each grading score for histiocytic changes, fibroblastic changes, chronic inflammation, acute inflammation. The colors represent the grading score (scale from 0 to 4, where 0 = no pathology, 1 = minimal pathology, 2 = mild pathology, 3 = moderate pathology, 4 = severe pathology) shown in the key. There was no scarring, insertion trauma, necrosis or retinal damage in any of the implanted eight eyes. N = 32 locations from eight subjects.

Histopathology grading in implanted eyes for histiocytic changes, fibroblastic changes, chronic inflammation and acute inflammation is shown in Figure 13 as stacked counts using a five-point scale (0–4). The pilot subject (18_301) had a single location measured. For the remaining seven subjects, there were 31 locations overlying electrodes included from a potential maximum of 35 electrodes. At the time of histopathological assessment, there was no scarring, insertion trauma, necrosis or retinal damage seen in any of the eyes receiving an implant and chronic stimulation. In 81% of locations analyzed there was no histiocytic response, with 19% of locations showing a grade 1 histiocytic response. In 100% of locations analyzed, there was no fibroblastic response. In 72% of locations analyzed, there was no chronic inflammation, with 22% of locations showing a grade 1 response and 6% of locations showing a grade 2 response. For acute inflammation, 81% of locations showed no response, 13% had a grade 1 response and 6% had a grade 2 response. A PAS stain and GRAM stain in the implanted eye of a representative subject (19_306) that had an array implanted for 6.5 months did not reveal any fungus and or bacterial colonies present.

In fellow eyes, a total of 27 matching locations from seven subjects (excludes pilot subject 18_301) were assessed, with only two locations showing a grade 1 chronic inflammatory response, and all locations showing no histiocytic, fibroblastic or acute inflammatory response. Hence, overall, there was a limited, occasionally mild, tissue response to chronic implantation and stimulation in implanted eyes.

## 4 Discussion

### 4.1 Implant design, surgical approach and relevance for translation to human

A neuroprotective eye implant designed for use in people with residual central vision was developed and the study found it could be safely implanted in a large-eye preclinical model. The implant design features include positioning in peripheral retina in the suprachoroidal space (not directly adjacent to neural retina) with a simple, minimally invasive surgery. Surgical implantation went smoothly and subsequent imaging and functional testing showed there was no damage to the retina from surgical insertion. Furthermore, the device position was stable over time after an initial settling period where an expected thin fibrotic capsule develops, helping to hold the array in place, similar to that noted in the human suprachoroidal retinal prosthesis trials (Ayton et al., 2014; Petoe et al., 2021; Titchener et al., 2022; Petoe et al., 2024).

In the feline model, the prototype 1 “short” array did not extend past the ora serrata, hence the prototype 2 “long” array was preferred to assess the stimulation safety for both potential global (comparing implanted to fellow eye) and local (comparing location overlying and adjacent to electrodes) effects. Although the feline model is an excellent large-eye model with axial length of approximately 21 mm, it does have a difference in the thickness of the anterior and posterior sclera, which means that the insertion incision needs to be placed anteriorly. In human surgery, there is greater ability to position the insertion incision based on precise axial length calculations to ensure the implant is placed underlying the peripheral retina. Furthermore, as extraocular devices are known to provide efficacy for retinal neuroprotection, whether an implant extends over the ora serrata in human is not thought to be critical. Hence both prototypes would be suitable for human use. The peripheral array design is relevant to all diseases targeted (RP, AMD and glaucoma) as neuroprotection with electrical stimulation has a global effect (i.e., a centrally-place array is not required for macula diseases such as AMD) (Liu et al., 2022).

Further advantages of the surgical approach shown when extended to human studies includes the design to position the incision under the lateral rectus muscle, using the muscle over the Dacron patch as further wound support. Certainly, in two clinical trials with seven human recipients of a suprachoroidal retinal prosthesis with a similar incision position, there have been no reports of wound breakdown or infection (Ayton et al., 2014; Petoe et al., 2021; Petoe et al., 2024). Furthermore, the approach is designed with the position of the vortex veins in mind, ensuring that insertion of any device from the temporal aspect will not risk any damage to the vortex veins. Although only 38% of electrodes remained in compliance at study endpoint, the issue of markedly reduced electrode functionality over time only exists for the feline model, where a cable system over the skull to a backpack stimulation system is required. In human surgery, the cable is routed via an orbital notch around the side of the skull to a behind the ear stimulator-receiver system (similar to that used for a cochlear implant) and does not require routing over the neck region (that results in torsional forces on the cable system). Human participants in suprachoroidal retinal prosthesis studies have not had any ongoing discomfort or pain following surgical recovery periods and have had minimal loss of electrodes after 2 years (Ayton et al., 2014; Petoe et al., 2021; Petoe et al., 2024).

### 4.2 Safety of chronic low-level stimulation and relevance for translation to human

Safety assessments, both *in vivo* and histopathologically, showed that after up to 8.7 months of chronic low-level electrical stimulation, there was no damage to the retina in any subject. An experienced histopathologist and a senior researcher experienced in chronic stimulation studies determined there was no scarring, insertion trauma, or necrosis above or adjacent to electrodes. A mild histiocytic change, fibroblastic change, chronic inflammatory response and acute inflammatory response was seen in less than 30% of subjects. This is within expectations for a retinal implant; previous studies have shown a similar low level histopathological tissue response to passive and actively stimulated suprachoroidal implants (Villalobos et al., 2013; Nayagam et al., 2014; Leung et al., 2015; Abbott et al., 2018). Furthermore, the *in vivo* clinical assessments showed no functional or structural decline from implantation or stimulation. The indentation of the choroid near the tip of the array is akin to what has been observed previously (Abbott et al., 2018), and is not expected to hamper blood flow given its limited extent and that the network of surrounding choroid is not impinged.

In the large-eye feline model, there is unfortunately no easy and reliable model for slowly progressive retinal disease (Aplin et al., 2016), so it was not possible to assess efficacy in this model. However, due to the large volume of clinical efficacy data coming out using extraocular electrical stimulation approaches (Shinoda et al., 2008; Schatz et al., 2011; Bittner et al., 2018; Bittner and Seger, 2018; Parkinson et al., 2023; Stett et al., 2023), there is plenty of data to suggest that clinical trial of the implant should be the next step given the present study demonstrates clear safety data. Certainly, the recent systematic review of electrical stimulation in retinal diseases (Liu et al., 2021), suggests there is promising clinical data for the concept of electrical stimulation as a neuroprotective therapy and advises that large-scale randomized controlled clinical trials are required to tease out evidence-based stimulation protocols and provide reliable efficacy data. The previous extraocular clinical efficacy studies have used current amplitudes of 20–1,000 µA for short periods of 30–60 min weekly (Schatz et al., 2011; Bittner and Seger, 2018; Sinim Kahraman and Oner, 2020; Stett et al., 2023). The present study used continuous stimulation of 100 µA for up to 8-month, which is in the lower range of the previous studies. Using an implant underlying the retina, the electrical fields generated are closer to the target tissue (retinal cells) than electric fields generated by the extraocular devices. Hence, a potential advantage of the implant approach is that it may allow for an even safer device over a long period of time, where lower current levels are perhaps required. However, large-scale randomized controlled clinal trials are required to assess this hypothesis.

Suprathreshold stimulation of the peripheral retina with a MIRA array may generate phosphene perceptions in people with residual vision. However, it is very unlikely that stimulation in peripheral retina would interfere with or spread to affect central vision. Previous studies in humans with a centrally located suprachoroidal retinal prosthesis show derived phosphene maps correlate to the retinotopic layout of the array position (Sinclair et al., 2016; Titchener et al., 2023). Furthermore, previous cortical electrophysiology shows evidence that current spread is confined within the region of the electrodes being stimulated (Shivdasani et al., 2012). Additionally, suprathreshold stimulation is not always necessary for neuroprotective purposes as efficacy studies show a broad range stimulation parameters can be efficacious (Liu et al., 2021). If suprathreshold stimulation was used in a clinical trial of the MIRA device, phosphene perceptions could be managed by varying the stimulation levels and locations.

### 4.3 Reproducibility of the MIRA system ERG

The finding that the MIRA electrodes themselves reliably and reproducibly record an electroretinography signal to eliminate the need for corneal electrodes during an electroretinography session has advantages for potential long-term monitoring of how the efficacious the neuroprotective implant is, since less variable recordings improve the sensitivity to detect disease progression. Aside from improved detection of disease progression, the ability to use the MIRA electrodes for ERG recording gives clinicians in the future a potentially easy way to perform home monitoring if such a system became available.

### 4.4 Novel implantation system for preclinical chronic stimulation studies

For the previous retinal prosthesis safety studies (Nayagam et al., 2014; Abbott et al., 2018), an implanted bulla-connector system was used to connect the cable comprising platinum wires from the orbit to the feline bulla cavity, to a connected cable comprising stainless steel wires that traversed the skull, where it was screwed into place with the aid of a metallic clip, and exited via the neck tissue to connect with a back-pack system for powering the chronic stimulation experiments. This system was prone to failures at the bulla-connection location and there were also frequent instances of wounds, infection and inflammation affecting the skin and muscle tissue over the metallic skull screws and fixation hardware, which reduced the length of time chronic stimulation could be performed. Hence, for the current study, a new implant system was developed for the feline model with platinum wires routed all the way through to the connector for linking to the stimulation system in the backpack.

The novel implant system was found to be effective, and the subjects remained in good health over the period of up to 9 months implantation. The novel system had functional electrodes for longer (2 subjects with functional electrodes for 8 months) than the previous “bulla-connector” system (4 months stimulation maximum (Nayagam et al., 2014; Abbott et al., 2018)). This cable-to-stimulator system set up in a chronic preclinical model is advantageous for any future studies requiring a cable connection to a sensory system within the head (eye, cochlear, olfactory, etc.) with a key design feature including the redundancy of electrodes to compensate for electrode attrition. During years of working with this preclinical model, the surgical approach has progressed to optimize overall health outcomes. Furthermore, the design of the suprachoroidal arrays has resulted in high electrode yield over time with the fully-implantable clinical bionic eye system (Petoe et al., 2021; Petoe et al., 2024).

### 4.5 Advantages and limitations

The advantage of the current study is that it is assessing feasibility of surgical approach and chronic low-level stimulation safety using a well-characterized large eye model, that can be directly compared to previous studies of suprachoroidal implants including critical histopathological assessments that cannot be performed in human studies. The limitations of the study include the inability of the model to assess efficacy in a progressive disease, the fact that the presence of the tapetum in the feline model obscures clear visualization of the electrodes within the array with color fundus photos, the inability of the full-field ERG to measure localized retinal function, and the lack of imaging and histology data for the short array as it did not extend past the ora serrata. However, SDOCT scans did provide cross-sectional assessment of electrode position and SDOCT and histology provided a localized assessment of retinal ultrastructure for the long arrays. Cortical electrophysiology was not performed as the array was too peripheral to make recordings.

### 4.6 Conclusion

This minimally-invasive implantable approach to neuroprotective retinal stimulation can potentially be used in patients with residual vision as it does not interfere with natural vision nor evoke overlapping percepts with natural central vision. Furthermore, it is a potential alternative option to extraocular stimulation devices and may improve patient compliance by providing the advantage of easy set up in home environments for long-term, regular stimulation treatments. The safety data in this study provide the evidence and confidence for proceeding to a first-in-human clinical trial with the implant in a range of progressive, early-stage retinal and optic nerve diseases such as RP, AMD and glaucoma.

## Data Availability

The original contributions presented in the study are included in the article/Supplementary material, further inquiries can be directed to the corresponding author.

## Abbreviations

AMD, Age-related macular degeneration; ERG, Electroretinography; MIRA, Minimally invasive retinal-degeneration arrestor; RP, Retinitis pigmentosa; SDOCT, Spectral domain optical coherence tomography
